# Light Chain Deposition Disease and Light Chain Cast Nephropathy in a Patient With Multiple Myeloma and HIV Infection: A Case Report

**DOI:** 10.1155/crin/4508810

**Published:** 2026-02-08

**Authors:** Tamzyn Huisamen, Liezel Coetzee, Mogamat-Yazied Chothia

**Affiliations:** ^1^ Division of Nephrology, Department of Medicine, Faculty of Medicine and Health Sciences, Stellenbosch University and Tygerberg Hospital, Cape Town, Western Cape, South Africa, sun.ac.za; ^2^ Division of Anatomical Pathology, Department of Pathology, Faculty of Medicine and Health Sciences, National Health Laboratory Service and Tygerberg Hospital, Stellenbosch University, Cape Town, Western Cape, South Africa, sun.ac.za

**Keywords:** human immunodeficiency virus, light chain deposition disease, multiple myeloma, myeloma cast nephropathy

## Abstract

Kidney dysfunction is a common complication in multiple myeloma (MM), typically presenting with cast nephropathy as a single pattern of injury on kidney biopsy and infrequently occurs in people living with human immunodeficiency virus (PLHIV). A man in his 50s, newly diagnosed with HIV, who was admitted with community‐acquired pneumonia was found to have severe acute kidney injury requiring hemodialysis. Due to the lack of renal recovery, a kidney biopsy was performed, revealing fractured, periodic acid‐Schiff stain‐negative tubular casts with surrounding multinucleated giant cell reaction. Congo red staining was negative, but electron microscopy revealed granular subendothelial electron‐dense deposits. Immunofluorescence demonstrated lambda light chain restriction. A diagnosis of light chain deposition disease with concurrent light chain cast nephropathy was made. To the best of our knowledge, this is the first description of concurrent light chain cast nephropathy and light chain deposition disease in a PLHIV and MM and highlights the importance of a kidney biopsy in the evaluation of acute kidney injury in a PLHIV and concomitant myeloma.

## 1. Introduction

Kidney dysfunction is a frequent complication of multiple myeloma (MM). The three primary patterns of kidney injury resulting from the deposition of abnormal light chains within various compartments of the kidney include light chain cast nephropathy (LCCN), amyloidosis, and light chain deposition disease (LCDD) [1]. While most patients exhibit a single pattern of injury, rare cases with coexisting pathologies have been reported and may carry significant prognostic implications. Plasma cell dyscrasias may also occur in people living with human immunodeficiency virus (PLHIV); however, this has been infrequently described in the medical literature [2, 3].

Here, we present a case of MM complicated by acute kidney injury (AKI) in which both LCCN and LCDD were identified on kidney biopsy in a PLHIV.

## 2. Case Report

A man in his 50s presented to the emergency department with a 1‐week history of worsening dyspnea, fever, cough, and bilateral lower limb weakness. He denied alcohol or tobacco use and was not taking any chronic medications. On examination, he was in severe respiratory distress, necessitating intubation and mechanical ventilation. He was newly diagnosed with HIV, complicated by community‐acquired pneumonia, and was referred to the intensive care unit where he required mechanical ventilation for 6 days. Laboratory investigations revealed severe AKI. Hemodialysis was commenced for refractory hyperkalemia and pulmonary edema.

Clinical examination revealed a blood pressure of 108/93 mmHg and pulse rate of 138 beats per minute and he was apyrexial. Bilateral diffuse crackles were heard on auscultation, with normal heart sounds and a mildly laterally displaced apex beat. Following extubation, neurological examination in the general medical ward revealed that the patient was alert and fully oriented to time, place, and person; however, power was 0/5–Medical Research Council grading of muscle power: 0 = No visible or palpable movement; 1 = Twitch or flicker of contraction felt/seen; 2 = Can move the limb, but only when gravity is removed; 3 = Lifts the limb fully against gravity, no resistance tolerated; 4 = Full range against gravity plus resistance, but strength is reduced relative to normal; and 5 = Normal power––in bilateral hip flexion and knee extension. No muscle wasting was present. Plantar and dorsiflexion were 3/5 bilaterally. Upper limb power was normal. He had absent reflexes and reduced tone in both his lower limbs. Sensation in the upper and lower limbs remained intact, and he had normal bladder and bowel function. The abdominal examination was normal, and there was no bone tenderness. Urine dipsticks showed 4+ protein without blood. Urine protein excretion was 8.16 g/L. Unfortunately, the urine protein‐to‐creatinine ratio could not be determined due to a laboratory error in the measurement of the urine creatinine concentration. As the patient subsequently became anuric, no further urine samples were available for analysis.

Laboratory findings (Table 1) showed mild anemia and leukocytosis. Using the total serum protein and serum albumin, the calculated serum albumin‐to‐globulin ratio was normal at 1.56 (normal range: 1.1–2.5). Severe AKI accompanied by marked hyperkalemia was observed. No prior measurements of kidney function were available before this presentation. Total serum calcium levels were within the normal range, and mild rhabdomyolysis was indicated by an elevated creatine kinase. Respiratory viral panel testing was positive for both rhinovirus and adenovirus, while GeneXpert testing for *Mycobacterium tuberculosis* was negative. Kidney ultrasound showed normal renal sizes with no evidence of obstruction.

**TABLE 1:** Laboratory results.

Variable	Reference range	Result
Sodium	135–145 mmol/L	125
Potassium	3.5–5.1 mmol/L	8.4
Urea	2.1–7.1 mmol/L	18.1
Creatinine	64–104 μmol/L	593
eGFR	> 90 mL/min per 1.73 m^2^	8
Total protein	60–78 g/L	59
Albumin	35–52 g/L	36
Calcium	2.15–2.5 mmol/L	2.32
Magnesium	0.63–1.05 mmol/L	1.17
Phosphate	0.78–1.42 mmol/L	3.07
Alanine transaminase	10–40 U/L	45
Leucocyte	3.92–10.40 × 10 ∗ 9/L	12.74
Hemoglobin	13.0–17.0 g/dL	9.4
Platelets	171–388 × 10 ∗ 9/L	294
C‐reactive protein	< 10 mg/L	80
Creatine kinase	20–200 U/L	2321
CD4 count	332–1642 cells/μL	321

Abbreviations: CD4, cluster of differentiation four; eGFR, estimated glomerular filtration rate; HIV, human immunodeficiency virus.

**FIGURE 1:**
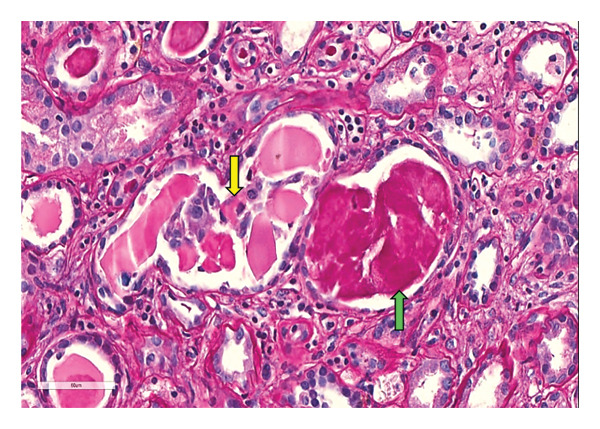
Periodic acid‐Schiff‐stained sections showed pale staining myeloma casts (yellow arrow) compared to darker staining Tamm–Horsfall proteinaceous casts (green arrow).

**FIGURE 2:**
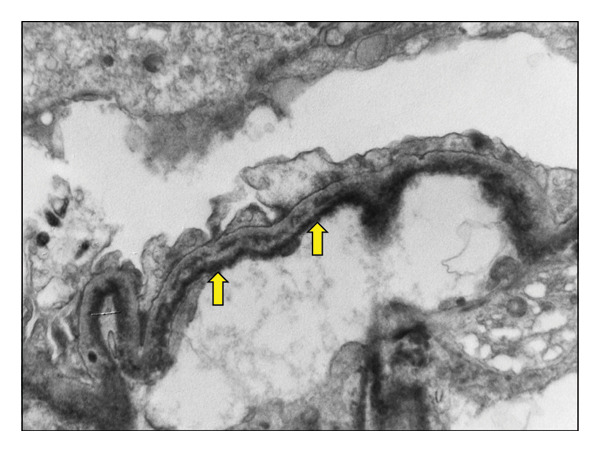
Ultrastructural evaluation showed flocculent to granular electron dense material in the subendothelial locations of the glomerular loops (yellow arrows).

Following 2 weeks of acute hemodialysis support, the patient failed to show renal recovery. A kidney biopsy was subsequently performed, yielding a total of 20 glomeruli: five on light microscopy, of which one was globally sclerosed, and 15 on electron microscopy (EM). The periodic acid–Schiff (PAS) stain revealed negative tubular casts with fracture planes and multinucleated giant cells (Figure 1). Congo red staining was negative for amyloid deposition. The interstitial compartment and vascular structures were unremarkable. EM demonstrated flocculent to granular electron‐dense material along the subendothelial regions of the glomerular basement membranes (Figure 2). Direct immunofluorescence (IF) confirmed lambda light chain restriction predominantly within the myeloma casts.

A diagnosis of LCDD and LCCN was established. Following the kidney biopsy, additional investigations were undertaken to evaluate for an underlying plasma cell dyscrasia. Serum‐free lambda light chains were markedly elevated (1540 mg/L), and kappa light chains were low (19.6 mg/L) with a kappa‐to‐lambda ratio of 0.01 (normal range during kidney dysfunction: 0.37–3.1). Serum protein electrophoresis demonstrated immune paresis with a monoclonal peak. Immunofixation revealed two free lambda monoclonal proteins but was too small to quantify. Bone marrow aspirate demonstrated atypical plasma cells, including large and multinucleated forms, with occasional prominent nucleoli. Immunohistochemical staining with CD138 was positive in some plasma cells, while multiple myeloma oncogene 1 (MUM1) stain was positive in all. The plasma cells were kappa‐negative and lambda‐positive, comprising approximately 20% of all nucleated marrow cells. No high‐risk chromosomal changes associated with MM were found on fluorescence in situ hybridization (FISH) including 1p deletion, 1q gain, 17p deletion, and immunoglobulin heavy chain rearrangement.

His neurological findings prompted a whole‐spine magnetic resonance imaging, which revealed only lower lumbar degenerative changes without evidence of a plasmacytoma. Lumbar puncture revealed a clear and acellular cerebrospinal fluid with a markedly elevated protein of 2.9 g/L (normal: < 0.45 g/L) and normal glucose level of 2.9 mmol/L, which was ∼60% of the plasma glucose value. Flow cytometry was not performed. Nerve conduction studies showed a symmetrical axonal neuropathy. A nerve biopsy had been planned; however, the patient passed away before the procedure could be performed.

## 3. Discussion

MM is a hematological malignancy marked by the clonal expansion of plasma cells within the bone marrow, often resulting in systemic complications, including kidney dysfunction [4]. Kidney dysfunction is one of the most common presenting features and occurs in approximately 20%–50% of the patients at diagnosis [5]. Kidney injury in MM caused by the deposition of monoclonal free light chains is heterogenous and primarily manifests as three main patterns of injury: (1) LCCN, (2) amyloidosis, or (3) LCDD [1]. MM usually presents with a single pattern of kidney injury with each of these pathologies having distinct clinical manifestations, histopathological features, and prognostic significance [6, 7].

The specific pattern of kidney injury is thought to be determined by the physiological properties of the circulating monoclonal light chains involved and their various local effects within the kidney [1]. While each pattern may independently lead to progressive kidney dysfunction, the coexistence of multiple monoclonal‐related kidney pathologies is considered uncommon and has been associated with worse clinical outcomes [8, 9]. To the best of our knowledge, the simultaneous occurrence of LCCN and LCDD in a PLHIV has not been previously described.

LCCN is the most frequent kidney manifestation of MM and is recognized as a myeloma‐defining event [10]. In LCCN, the overproduction of free light chains results in saturation of the resorptive capacity of the proximal tubules, leading to intratubular precipitation with uromodulin in the distal nephron. This results in obstructive cast formation, tubular atrophy, and interstitial inflammation [10]. Histologically, these casts are PAS‐negative with fracture lines and are often associated with a giant cell reaction [11].

In contrast, LCDD is characterized by granular deposits of monoclonal light chains along glomerular and tubular basement membranes. Kappa light chains are implicated in most cases (∼75%) [10]. Histology commonly reveals nodular mesangial sclerosis. IF demonstrates monoclonal light chain restriction (*κ* or *λ*), while EM shows granular electron‐dense deposits along basement membranes [12].

When LCCN and LCDD occur simultaneously, prognosis is significantly poorer. Studies have shown that patients with dual pathology have poorer outcomes [8, 9]. In a study of 87 patients with myeloma‐related kidney disease due to LCDD and LCCN, patients were grouped as having both LCDD and LCCN, LCCN only, or LCDD only. Prognosis in patients with both LCDD and LCCN was similar to those with LCCN alone but worse than in patients with LCDD alone. Kidney survival, after censoring for death, did not differ between groups. AKI at the time of biopsy was an independent predictor of poor renal survival, whereas older age, need for dialysis, or a serum creatinine ≥ 442 μmol/L at biopsy predicted overall reduced survival [8]. Our case aligned with these findings.

The intersection of MM and HIV infection remains poorly described, especially in low‐ and middle‐income countries, where access to diagnostic resources and comprehensive kidney pathology services may be limited. Although plasma cell dyscrasias in PLHIV have been previously sporadically reported, the overall incidence appears to be increasing, likely representing the improved survival rates in PLHIV given the improved availability and promotion of antiretroviral therapy. Tubular casts composed of plasma proteins can occasionally be seen in HIV‐associated nephropathy; however, they typically stain PAS‐positive and are polyclonal in nature but may sometimes mimic myeloma casts [2].

PLHIV who develop MM often present at a younger age and display distinct clinical features, including fewer osteolytic lesions, a higher proportion of monoclonal IgG proteins, and less severe kidney dysfunction compared with HIV‐negative individuals [13]. Kidney complications related to monoclonal gammopathies in PLHIV are rarely described in the literature, posing a challenge to the timely diagnosis and appropriate treatment strategies in this population group.

In our patient, the coexistence of LCCN and LCDD in the setting of HIV infection represents a rare pathological finding. The kidney biopsy demonstrated both obstructive casts and monoclonal light chain deposits, confirming dual pathology. This case emphasizes the importance of a kidney biopsy when evaluating AKI of unknown cause in PLHIV.

Regarding the lower limb weakness, spinal cord compression from a plasmacytoma was initially suspected; however, spinal MRI excluded this possibility. Nerve conduction studies––performed 2 weeks after admission to the intensive care––demonstrated an axonal pattern of peripheral nerve injury. Although myeloma‐related neuropathy occurs in approximately 1%–8% of the patients, it is typically sensory and associated with kappa light chains in about 65% of the cases [14]. In contrast, our patient exhibited predominantly motor weakness and lambda light chains. Intensive care‐acquired critical illness weakness was considered unlikely because the onset of the weakness occurred 1 week prior to hospitalization, only the lower limbs were affected, and he did not receive any neuromuscular blocking agents and had a brief stay––six days––in the intensive care [15]. We, therefore, concluded that the most likely contributors to the peripheral neuropathy were uremic polyneuropathy [16] and/or HIV‐associated neuropathy [17].

In conclusion, to the best of our knowledge, this is the first description of concurrent LCCN and LCDD in a PLHIV and MM and highlights the importance of a kidney biopsy in the evaluation of kidney dysfunction in a PLHIV and concomitant myeloma.

## Author Contributions

Writing–original draft: Tamzyn Huisamen; writing–review and editing: Tamzyn Huisamen, Liezel Coetzee, and Mogamat‐Yazied Chothia; conceptualisation: Liezel Coetzee and Mogamat‐Yazied Chothia.

## Funding

The authors received no financial support for the research, authorship, and/or publication of this article.

## Consent

The patient gave written informed consent to publish, and the Health Research Ethics Committee (HREC) of Stellenbosch University granted permission to publish this case report (HREC reference number: C25/09/037; Project identification: 34561).

## Conflicts of Interest

The authors declare no conflicts of interest.

## Data Availability

Data used to support the findings of this study are available on request from the corresponding author.
